# Calcium Signalling in Plant Biotic Interactions

**DOI:** 10.3390/ijms19030665

**Published:** 2018-02-27

**Authors:** Didier Aldon, Malick Mbengue, Christian Mazars, Jean-Philippe Galaud

**Affiliations:** Laboratoire de Recherche en Sciences Vegetales, Universite de Toulouse, CNRS, UPS, 24, Chemin de Borde-Rouge, Auzeville, BP 42617, 31326 Castanet-Tolosan, France; aldon@lrsv.ups-tlse.fr (D.A.); malick.mbengue@lrsv.ups-tlse.fr (M.M.); mazars@lrsv.ups-tlse.fr (C.M.)

**Keywords:** biotic stress responses, calcium, calcium signature, calmodulin, CMLs, CDPKs, plant immunity, symbiosis

## Abstract

Calcium (Ca^2+^) is a universal second messenger involved in various cellular processes, leading to plant development and to biotic and abiotic stress responses. Intracellular variation in free Ca^2+^ concentration is among the earliest events following the plant perception of environmental change. These Ca^2+^ variations differ in their spatio-temporal properties according to the nature, strength and duration of the stimulus. However, their conversion into biological responses requires Ca^2+^ sensors for decoding and relaying. The occurrence in plants of calmodulin (CaM) but also of other sets of plant-specific Ca^2+^ sensors such as calmodulin-like proteins (CMLs), Ca^2+^-dependent protein kinases (CDPKs) and calcineurin B-like proteins (CBLs) indicate that plants possess specific tools and machineries to convert Ca^2+^ signals into appropriate responses. Here, we focus on recent progress made in monitoring the generation of Ca^2+^ signals at the whole plant or cell level and their long distance propagation during biotic interactions. The contribution of CaM/CMLs and CDPKs in plant immune responses mounted against bacteria, fungi, viruses and insects are also presented.

## 1. Introduction

Like all living organisms, plants face environmental challenges that can be either of a biotic nature such as interactions with pathogens (e.g., bacteria, fungi, oomycetes, viruses, insects) or of an abiotic nature such as drought, soil salinity, air pollution, extreme temperatures and mechanical injury [1]. These adverse conditions often limit growth and productivity of crops worldwide. The expected global temperature elevation in the coming years and associated climate modifications are creating ever-greater challenges for agriculture [2,3]. To adapt to adverse growth conditions, plants must be able to detect the nature and strength of environmental stimuli, interpret them and activate appropriate physiological responses [3]. Among signalling elements that are involved in plant stress responses and particularly during immune responses to pathogens, reactive oxygen species (ROS) and Ca^2+^ ions are among the earliest actors that coordinate plant adaptive responses [4,5,6,7]. The oxidative burst was first described in 1983 following Potato infection by the oomycete *Phytophtora infestans* [8], whereas the importance of Ca^2+^ signalling in plant immunity was reported in tobacco following *Pseudomonas syringae* inoculation in 1990 [9]. A close connection was then established between ROS and Ca^2+^ signalling pathways in plant immunity [10].

**Figure 1:**
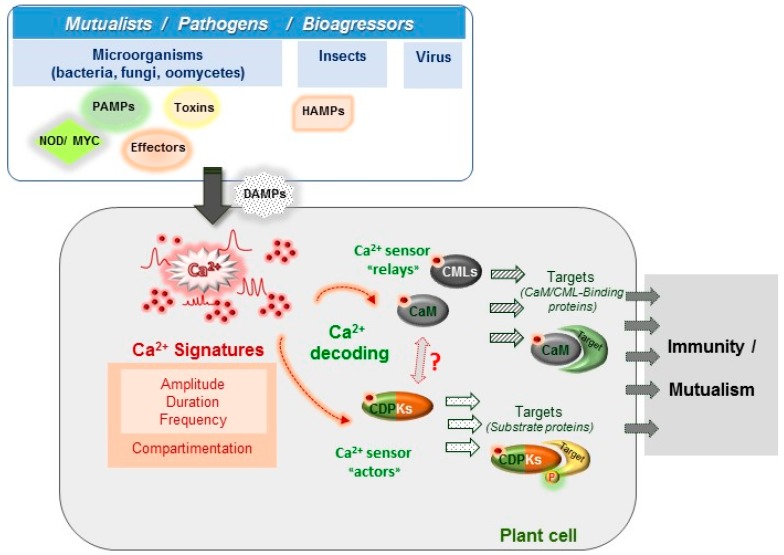
Key steps in Ca^2+^ signaling pathways during plant biotic interactions. Plants are exposed to diverse microorganisms, pests or other aggressors leading to beneficial or detrimental interactions. Plant cells possess a large repertoire of sensors that allow to perceive, discriminate and transduce different signals during plant immunity (Pathogen-Associated Molecular Patterns (PAMPs) , effectors, toxins, Damage-Associated Molecular Patterns (DAMPs) or Herbivory-Associated Molecular Patterns (HAMPs)) or during the interaction with mutualistic organisms (Nod and Myc Factors). In response to different stimuli, the earliest steps rely on specific cytosolic Ca^2+^ rises termed calcium signatures occurring in the cytosol and in organelles, including nucleus (Section 2.1). These calcium signatures differ by their spatio-temporal properties and encode a first layer of specificity. A second layer of specificity, relies on the decoding of these calcium transients (Section 3). Ca^2+^ binds to a plethora of sensors such as calmodulin (CaM), CaM-like proteins (CML), calcium-dependent protein kinases (CDPK) that activate target proteins either by direct binding or through phosphorylation (P).

In this review, we will focus on the importance of Ca^2+^, a ubiquitous and versatile second messenger [11], in plant biotic interactions. To become informative, the Ca^2+^ message needs to be decoded and relayed in order to activate the appropriate cell response and this is carried out by Ca^2+^-binding proteins termed Ca^2+^ sensors [12]. The complex spatiotemporal patterns of Ca^2+^ changes at cellular and tissue levels (frequency, amplitude, and distribution within the cell) are proposed to carry information and are denoted as the Ca^2+^ signature [13,14] (Figure 1). The Ca^2+^ signature encodes a first layer of specificity and will be considered first, with a particular emphasis on how methods to monitor Ca^2+^ signatures have evolved and brought new information. Ca^2+^-binding proteins and their downstream targets provide a second layer of specificity. Most Ca^2+^-binding proteins are characterized by the presence in their sequence of the canonical Ca^2+^-binding motif called the EF-hand [15]. For example, the plant model *Arabidopsis thaliana* encodes at least 250 EF-hand-containing proteins [16]. This number is much higher than in mammals and notably, the majority of plant Ca^2+^ sensors do not have homologs in others organisms [17,18]. However, only about half of them have been considered as Ca^2+^ sensors [17]. These plant Ca^2+^ sensors are classified into four major groups: the calcineurin B-like (CBL), the Ca^2+^-dependent protein kinases (CDPK), the calmodulin (CaM) group and its closely related group, the Calmodulin-like protein (CML) family [17,19]. Calmodulin (CaM) is one of the most studied eukaryotic proteins and has been shown to interact with and modulate the activity of numerous target proteins [20]. Plants also possess a remarkable repertoire of CaM-related proteins termed CMLs (7 CaM and 50 CMLs genes in *Arabidopsis*) that are not present in animals, as is also the case for CBLs and CDPKs (ten and 34 genes in Arabidopsis, respectively) [21,22]. To date, the roles of most of these Ca^2+^ sensors remain unknown but recent studies have pointed out the roles for some of them in physiological processes associated with development, abiotic plant stress responses and plant immunity [23,24,25]. Here, we present recent and relevant data about CaM, CMLs and CDPKs and their involvement in plant responses to various biotic stresses.

## 2. Ca^2+^ Signatures as the Earliest Signalling Events in Plant-Organism Interactions

### 2.1. Discovering the Importance of Ca^2+^ Signalling in Biotic Interactions

Free Ca^2+^ is a universal second messenger, and increases in cytosolic Ca^2+^ concentration are among the earliest signalling events occurring in plants challenged with mutualistic or pathogenic partners. If we consider plant-pathogen interactions, the plant immune system is schematically organized as a two-tiered system composed of Pathogen-Associated Molecular Patterns (PAMP)-Triggered Immunity (PTI) and Effector-Triggered Immunity (ETI) [26]. Activation of PTI enhances overall plant defence and protects plants from subsequent pathogen attack [27] while activation of ETI often culminates in a localized programmed cell death (PCD) also referred to as the Hypersensitive Response (HR), which blocks pathogen invasion. These immune layers also differ in their Ca^2+^ signature. For instance, PTI activation involves Ca^2+^ transients that returns to basal levels within minutes [28] whereas ETI is associated with a prolonged cytosolic Ca^2+^ increase that can last for hours [29]. The generic Ca^2+^ channel blocker lanthanum strongly impairs the immune responses associated with both types of plant immunity [29,30], thereby placing Ca^2+^ signals and their decoders at the centre of immune signalling pathways.

Ca^2+^ elevations during immune signalling are critical for the control of gene reprogramming which is required to mount the adequate responses (e.g., symbiosis or defence). Such widely acknowledged importance of these Ca^2+^ variations in plant signalling has been made possible through the emergence in the early 1990s of tools allowing the monitoring of the dynamics of Ca^2+^ changes in plant cells. The pioneering work of Trewavas’s group and his collaborators demonstrated the occurrence of Ca^2+^ variations in plant cells using the fluorescent Fluo3 Ca^2+^ indicator and caged Ca^2+^ or IP3 as triggers [31]. His group then opened a new research avenue mainly through the application, for the first time in plants, of the non-invasive, organelle-addressable and highly dynamic Ca^2+^ probe aequorin [32]. The use of aequorin allowed tremendous progress in our current understanding of how Ca^2+^ controls plant adaptive responses. In the seminal paper reporting the use of aequorin in plants, Knight et al. established for the first time that biotic stimuli consisting of various yeast preparations were able, like abiotic stimuli, to trigger Ca^2+^ signals in tobacco seedlings [32]. This result suggested that the Ca^2+^ cation should be taken into consideration as an important second messenger in studies dealing with plant-microbe or plant-pest interactions. Due to the numerous studies available in the literature, we will focus in this section, more specifically on several well-documented examples illustrating how exploiting the diversity of Ca^2+^ probes has contributed to increasing our knowledge on the role of Ca^2+^ during plant immunity, symbiosis and response to herbivory attacks.

Chandra and Low pointed out the advantages of the aequorin probe versus other available Ca^2+^ sensors [33]. Using a suspension of aequorin-transformed tobacco cells, they reported that oligogalacturonic acid, a pectin-derived component, was able to induce plant defence and to generate a Ca^2+^ response followed by a delayed oxidative burst [34,35]. Using a pharmacological approach, they demonstrated that the oxidative burst was fully dependent on the Ca^2+^ response. This result was among the first to suggest a possible role of Ca^2+^ transients in the ROS-mediated defence signalling pathway [33]. At the same time, Mithoefer et al. established a similar link between cytosolic Ca^2+^ increases and defence responses using chitotetraose, a fungal cell wall component and β-glucans. A sustained high Ca^2+^ concentration response was observed in the case of β-glucans while this was not the case with the chitotetraose [36]. The sustained Ca^2+^ increase generated after β-glucan application was linked to the increase of a major soybean phytolalexin glyceollin [36]. One year later, Blume et al. demonstrated that a *Phytophtora sojae*-derived peptide (Pep 13) elicited a biphasic Ca^2+^ variation due to an influx of extracellular Ca^2+^ in parsley cells stably expressing aequorin. Only the sustained Ca^2+^ variation reaching a concentration around 250–300 nM could be associated with ROS and phytoalexin production [37]. Interestingly, stimuli that triggered only the first transient peak were unable to stimulate phytoalexin production [37]. Similarly, the use of *Nicotiana plumbaginifolia* cells stably expressing cytosolic aequorin challenged with the *Phytophtora cryptogea* elicitin “cryptogein” allowed us to demonstrate that oligogalacturonic acids and cryptogein each elicit a very specific cytosolic Ca^2+^ response [38]. After a lag phase of 1–2 min, the cryptogein-induced Ca^2+^ response appeared as a biphasic Ca^2+^ increase with a first transient peak lasting 10 min followed by a sustained increase. Suppression of this sustained Ca^2+^ phase using the Ca^2+^ channel blocker lanthanum chloride just after the first Ca^2+^ transient, resulted in the suppression of MAPK activation, accumulation of *PAL* and *hsr203j* immune-related gene transcripts and induced cell death [38].

Most of the above studies were performed with elicitors of various nature and origin coming from fungal or bacterial pathogens such as Microbe Associated Molecular Patterns (MAMPs) or compounds derived from the activity of pathogens on plant cell walls, the Damage-Associated Molecular Patterns (DAMPs) [39,40]. Similarly, elicitors can originate from herbivores and are named Herbivore Associated-Molecular Patterns (HAMPS). They are found in oral secretions or oviposition fluids of insects (for a review see [41]). HAMPs have also been shown to induce cytosolic Ca^2+^ variations, for example in lima bean leaves incubated with the fluorescent Ca^2+^ probe Fluo3-AM or in soybean cell cultures expressing cytosolic aequorin. *Spodoptera littoralis* larvae bites elicited a Ca^2+^ response on the margin of the leaves within a delimited region of 30 to 200 µm from the bite zone whereas several components from the larvae regurgitate such as linolenoyl-L-Glutamine or volicitin were able to elicit Ca^2+^ transients in soybean cells [42]. These Ca^2+^ transients are correlated with early membrane depolarization/hyperpolarization events that are known to be linked to plant defence responses through systemin synthesis, ROS and jasmonate signalling (for a review see [43]).

These few examples showing that different elicitors trigger different Ca^2+^ variations reinforced the concept of the Ca^2+^ signature [13,14]. This concept postulates that a specific Ca^2+^ variation is defined by its form, amplitude, frequency, duration, spatial localization and the Ca^2+^ pool involved. All these parameters are tightly linked to the nature and strength of the stimulus perceived by the cell. Subsequent studies performed on guard cells largely corroborated this concept. Artificial modulation of the frequency, transient number of spikes, duration and amplitude of Ca^2+^ oscillations were found to control the degree of long-term steady-state stomata closure [44]. Other findings supporting the Ca^2+^ signature concept come from work performed with *Medicago truncatula* plants expressing the Ca^2+^ probe cameleon YC2.1. Using a pharmacological approach modulating Ca^2+^ homeostasis, the authors were able to demonstrate a link between the bacterial Nod factor-induced Ca^2+^ oscillations and the activation of some selected nodulation markers genes. For example, the induction of the *ENOD11 nodulin* gene required about 30 consecutive Ca^2+^ spikes [45].

### 2.2. Monitoring Ca^2+^ Transients at the Whole Tissue or Organism Level

Most of the initial studies on Ca^2+^ variations in plant immunity were performed with elicitors and cultured cells. Although convenient, this model cannot inform us about the downstream events activated during genuine plant-microorganism or plant-pest interactions, such as cell-to-cell communication leading to long distance and systemic signalling. Pathogen elicitors only mimic the recognition step when surface motifs of pathogens are recognized by pattern recognition receptors (PRRs) of the host [7]. In addition to this recognition of surface motifs, pathogens also inject effectors into the plant cell to modulate plant defence responses [46]. Therefore, if we consider pathogenic bacteria, both compatible and incompatible strains possess the same PAMPs but in the first case the interaction leads to plant susceptibility while in the second case the interaction may lead to plant resistance through the execution of the HR due to effector recognition by plant resistance (R) genes [47,48]. Aequorin allows long kinetic studies due to its stability in the cell [49]. It has enabled the analysis of Ca^2+^ signalling in whole plants challenged with a microorganism (pathogen, symbiont) or insect, in a non-destructive manner. One of the first examples of such studies, was the monitoring of Ca^2+^ response in Arabidopsis leaves stably expressing aequorin under control of the 35S promoter challenged either with the compatible strain of *Pseudomonas syringae* pv. *tomato* DC3000 or with incompatible strains carrying the avirulence genes *avrRPM1* or *avrB*, both detected by the resistance Arabidopsis RPM1 protein [29]. In this study, the authors showed that the compatible strain elicited a transient Ca^2+^ peak lasting about 10 min and peaking between 8–12 min whereas the incompatible strains avrRPM1 and avrB induced an additional delayed peak giving a maximal increase at around 105 ± 10 and 137 ± 7 min, respectively [29]. This second peak was dependent on a functional type III secretion apparatus because DC3000 mutants in this secretion system did not induce this second Ca^2+^ response. In addition, Arabidopsis *rpm1* mutant failed to generate the second Ca^2+^ peak and the HR. Thus, the aequorin technology allowed, in this specific case, to make a clear correlation between the delayed Ca^2+^ peak and the gene for gene interactions responsible for the HR response.

In symbiotic interaction studies, the use of Oregon-dextran dye injected in root hair cells by iontophoresis demonstrated that two important genes in the rhizobia symbiosis were involved upstream of the Ca^2+^ spikes [50]. Using the same fluorescent Ca^2+^ probe, this group demonstrated two years later that spatiotemporal Ca^2+^ spiking studies were helpful in understanding the regulation of nod gene expression. Indeed, they showed that strains differing by their ability to produce Nod factor could be differentiated by the Ca^2+^ oscillations they induce. Thus, two strains derived from the same parent displayed large differences in the kinetics of Ca^2+^ spiking; *Rm1021* triggered a robust Ca^2+^ spiking after a lag phase of 10–15 min in more than 50% of the root hair cells whereas *Rm2011* did not [51]. The new generation of available genetically encoded Ca^2+^ probes [52] facilitated the measurement of Ca^2+^ responses at the organ and cell level during symbiotic relationships with rhizobia and mycorrhizal fungi. Kosuta et al. [53] were able to compare and discriminate the two types of symbiosis through their induced Ca^2+^ oscillations. They demonstrated that the two symbiotic pathways require both DMI1 and DMI2 for both the establishment of functional symbiosis and also for the generation of Ca^2+^ oscillations. This result indicates that Ca^2+^ oscillations are also required for plant colonization by rhizobia or mycorrhizal fungi. Interestingly, the symbiotic fungus induced a Ca^2+^ oscillation that differs from the Nod-factor-induced Ca^2+^ oscillations by its amplitude and its periodicity [53]. In the bacterial symbiotic model, the use of nucleoplasmin-tagged cameleon (NupYC2.1) Ca^2+^ probe demonstrated a regular nuclear Ca^2+^ spiking in *Medicago* root hairs challenged with Nod Factors [54].

### 2.3. Systemic Ca^2+^ Signalling during Plant Defence

The whole plant approach allowed researchers to detect long-range Ca^2+^ waves, but most of these studies were performed in response to abiotic stimuli and were exploiting either cameleon derivatives such as the YCNano-65 FRET sensor [55,56] or Bioluminescence Resonance Energy Transfer (BRET) [57]. Using the BRET system, Ca^2+^ waves propagating from the root to the shoot were reported in response to salt [57]. A similar study reported comparable Ca^2+^ waves and that the Two-Pore Channel (TPC1) vacuolar plant Ca^2+^ channel appeared to be a main player in this spreading [55]. To our knowledge, in response to biotic stimuli, the most significant data displaying systemic Ca^2+^ responses have come from plant–insect interactions [58]. Wounding or caterpillar feeding on a specific Arabidopsis leaf was found to elicit Ca^2+^ signals on the neighbouring leaves. Interestingly, TPC1 appeared to be involved in the systemic propagation of the Ca^2+^ wave but not to be involved in the generation of the primary Ca^2+^ transient observed at the feeding point [58]. The recent emergence of intensity-based Ca^2+^ sensors has made it possible to evaluate the spatio-temporal specificity of immune responses as well as the direction in which the PAMP-induced Ca^2+^ waves propagate [59]. Using R-GECO as an intensity-based Ca^2+^ sensor, Keinath et al. studied Ca^2+^ responses induced by Pathogen-Associated Molecular Patterns (PAMPS) such as flagellin and chitin [60]. They showed that flagellin, known to induce monophasic Ca^2+^ variations in cultured cells when Ca^2+^ changes are monitored with aequorin, was also able to induce Ca^2+^ variations in roots. They localized these variations to the elongation zone where callose deposition takes place and immune-responsive genes are upregulated supporting a clear correlation between Ca^2+^ changes and defence responses [60].

Overall, these few examples highlight the complexity of Ca^2+^ signalling and illustrate how monitoring Ca^2+^ signals at the cell tissue or whole plant level and the use of alternative Ca^2+^ probes from the available panel can be very helpful to decipher both immune and symbiotic responses.

## 3. Ca^2+^ Decoding Processes and Plant Immunity

The information encrypted by Ca^2+^ signals previously described (Section 2.1) needs to be decoded and relayed by Ca^2+^ binding proteins in order to be converted into biological responses (Figure 1). Following Ca^2+^ binding, sensors undergo conformational changes leading either to the regulation of their own catalytic activity or to their interaction with target proteins [61]. In plants, Ca^2+^ sensors are classified into three sub-groups including calmodulin (CaM) and calmodulin-like proteins (CMLs), Ca^2+^-dependent protein kinases (CDPKs) and calcineurin B-Like proteins (CBLs) (for reviews see [62,63,64,65]). In this review, we will focus and present information on CaM, CMLs and CDPKs, due to increasing evidence for their involvement in plant immunity. The importance of Ca^2+^ signalling in symbiosis has been recently reviewed, as well as the function of CBLs, which are mainly related to the regulation of membrane proteins involved in plant development, nutrition and abiotic stress [65].

### 3.1. CaM and CaM Binding Proteins in Plant Immune Responses

CaM is the prototype of a Ca^2+^-binding protein and is found in all eukaryotic cells. CaM acts as part of a Ca^2+^ signalling pathway by modifying its interactions with various CaM-binding proteins (CaMBP). Genome analysis of the model plant *A. thaliana* revealed the presence of 3 distinct CaM isoforms (encoded by seven genes) [17,66]. Until now, most of our knowledge about CaM mainly relies on the identification of the repertoire of CaMBPs [67]. During the last decade, the development of large scale yeast two-hybrid protein array screens, as well as proteomic analyses, increased our knowledge of CaM targets [67,68,69]. We can conclude from these analyses that CaM exerts a regulatory role on a wide variety of cellular processes by modulating the activity of various proteins such as channels, enzymes and transcriptional regulators [5,68,70,71]. Although many CaM-binding transcription factors (TFs) have been identified in diverse families of DNA binding proteins including plant-specific TFs [5,72], in most cases, the biological significance of the interaction with CaM remains to be elucidated.

CaMBPs with predicted or demonstrated functions during plant immunity have been reported [5,24]. For example, several pathogen-induced CaM-binding TFs have been associated with plant defence responses by acting on homeostasis regulation by salicylic acid (SA), a defence-associated hormone in plants [63,73]. The production of SA in *A. thaliana* infected cells is enhanced by up-regulation of the expression of *ICS1* (*Isochorismate synthase 1*) and *EDS1* (*Enhanced Disease Susceptibility 1)* genes and the expression of *ICS1* and *EDS1* is positively and negatively controlled by CBP60g and CAMTA3/AtSR1, respectively, two CaM-binding TFs [73,74,75].

CBP60g belongs to a plant-specific DNA-binding protein family comprising eight members in Arabidopsis [74,76,77]. The *cbp60g* knock-out mutant exhibits defects in pathogen-induced SA accumulation and shows enhanced susceptibility to the *Pseudomonas syringae* phytopathogenic bacteria [74]. A variant of CBP60g unable to bind CaM, does not restore SA production and defence and failed to complement the *cbp60g* mutant. This shows the importance of CaM in regulating CBP60g function and its contribution to plant immunity [74]. The role of other CBP60g-related proteins have been explored during plant–microbe interaction and to date, only CBP60a seems to contribute to plant immunity [77]. If CaM-binding is also crucial for the biological function of CBP60a, *cbp60a* mutations reduced *P. syringae* growth in planta, indicating that CBP60a acts as a negative regulator of immunity whereas CPB60g acts as a positive regulator [77]. Moreover, it was recently demonstrated that CML46 and CML47 negatively control SA accumulation in Arabidopsis and that this effect is genetically linked and additive to that of CBP60a [78]. Altogether, these data indicate a complex regulation of SA-dependent processes involving related TFs possessing (or not) CaM-binding activity and highlight the importance of the Ca^2+^-CaM/CML complex in the activation of immune responses.

The CBP60 transcriptional regulator family is not the only one regulated by Ca^2+^ and CaM. Indeed, the CaM-binding Transcription Activator (CAMTA) family certainly constitutes one of the most important Ca^2+^/CaM-regulated TF families in plants [79,80]. CAMTAs are key players in various plant biological processes including disease resistance and abiotic stress tolerance [81]. One of the pioneer studies concerned the functional analysis of CAMTA3, also known as AtSR1 (*Arabidopsis thaliana* Signal Responsive 1), in plant defence responses against pathogens [82] and its contribution as a negative regulator of the SA pathway [75]. Arabidopsis *camta3*/*Atsr1* Knock-Out mutants display elevated SA levels, high expression of *EDS1* and constitutive defence responses. CaM binding is crucial for the function of CAMTA3 in the control of SA production and defence [75]. Surprisingly, *camta3* mutants are more resistant to *P. syringae*, *Botrytis cinerea* and *Sclerotinia sclerotiorum* [82,83] but are more susceptible to insect attack (i.e., *Trichophusia*) [83]. These data suggest that CAMTA3 negatively regulates plant defence against biotrophic and necrotrophic pathogens by controlling endogenous levels of SA [75,82]. In addition, CAMTA3 was proposed to control plant resistance to herbivory insects through the regulation of glucosinolates metabolism [84,85].

Other studies revealed additional roles for CAMTA3/AtSR1 in defence. For instance, AtCAMTA3 negatively regulates plant immunity following PAMP recognition as well as non-host resistance to *Xanthomonas oryzae* [80]. CAMTA3 may negatively regulate PTI (PAMP-triggered Immunity) by targeting BAK1 [83]. More recently, a transcriptome comparative analysis using CAMTA modified transgenic plants has demonstrated activation of defence genes involved in both PTI and ETI (Effector-Triggered Immunity), suggesting that CAMTA could define an early convergence point in these two signalling pathways [86]. To date, it has not yet been elucidated how CaM can balance the activation of CBP60g and/or CAMTA3 upon pathogen attack with how the Ca^2+^ signatures are integrated in this transcriptional pathway. Efforts are being made in this perspective and dynamic mathematical models incorporating several parameters are being developed with the aim of predicting regulation networks according to the generated Ca^2+^ signatures [87,88].

Although the CaM contribution in plant immunity was mainly revealed by the identification of CaMBP, it is now clear how CaM plays a pivotal role in the fine tuning of immune responses by acting either as a positive or a negative regulator of defence responses. Identification of the whole set of CaMBPs is certainly not complete and other CaM-binding TFs including several members of TGA, WRKY, MYB and NAC families also contribute to plant immunity either positively or negatively [63,88,89]. For example, TGA3 and several WRKY transcription factors such as WRKY7 and WRKY53 can interact with CaM in a Ca^2+^-dependent manner [68] but the effects of this interaction on the physiological and/or biochemical function of these TFs remain unknown.

### 3.2. CMLs: Emerging Plant Ca^2+^ Sensors in Immunity

In addition to the presence of the typical CaMs, plants also possess a broad range of divergent forms of CaM called CaM-like proteins (CMLs, with 50 members in *A. thaliana*) [17,19]. Like CaMs, CMLs contain several EF-hand motifs, are predicted to bind Ca^2+^ ions and do not possess other known functional domains [17,19]. Whereas CaM encoding genes are uniformly and highly expressed, the expression patterns of *CMLs* vary according to plant developmental stages, tissues and environmental stimuli, indicating that each CML may have a specific role in plants [17]. Indeed, data on the physiological relevance of CMLs during plant physiology and more specifically in plant immunity have emerged during the last decade (reviewed in [6,90,91,92]).

Although we cannot rule out functional redundancy between members of the CaM/CML family, accumulating evidence indicate that deregulation of individual *CaM/CML* gene expression or loss of a CML function in mutated plants affect plant defence responses to various pathogens. The first report involving CMLs in plant defence came from gain-of-function experiments by overexpressing CMLs. The over-expression of soybean CMLs (i.e., SCaM-4/-5) in tobacco confers enhanced resistance to a wide spectrum of virulent and avirulent pathogens, including bacteria, fungi and viruses [93]. When constitutively expressed in *Arabidopsis*, SCaM-5 confers enhanced resistance to *Pseudomonas syringae* infection [94] whereas over-expression of SCaM-1 (a typical CaM) does not, which suggests that SCAM-4/-5 are specifically recruited in response to pathogens [94]. Although obtained in heterologous plant systems, these results suggest that CaM/CML isoforms are components of signal transduction pathways leading to disease resistance. Interestingly, it was later shown that the overexpression of SCaM-4 in soybean stimulated resistance to the oomycete *Phytophtora sojae* and to two necrotrophic fungal pathogens (*Alternaria tenuissima* and *Phomopsis longicolla*) supporting the idea that CaM/CMLs do take part in plant immune responses [95].

Loss-of- function genetic approaches also demonstrated roles in plant immunity for several CMLs. For instance, silencing *APR134* in tomato suppresses the hypersensitive response (HR), whereas overexpression of the APR134 orthologue from *Arabidopsis* CML43 stimulates the HR in response to an avirulent strain of *P. syringae* [96]. Similarly, *cml24* knockout in *Arabidopsis* impairs the HR response and reduces nitric oxide production following PAMP recognition [97]. Gain- and loss-of-function strategies were also developed on Arabidopsis CML8 and CML9 to evaluate their physiological function in plant stress responses. These two CMLs are positive regulators of plant defence against different strains of *P. syringae* [98,99,100]. CML9 was first described to be involved in plant responses to abiotic stress [101] and later shown to also contribute to plant immune responses [97]. The *cml9* mutants and *CML9* overexpressing lines exhibit enhanced or reduced susceptibility to virulent strains of *P. syringae,* respectively [98]. These phenotypes can be explained by alterations of flagellin-induced responses, including deposition of callose papillae and modifications of defence-related genes expression [98]. CML8 also takes part in plant immune responses against *P. syringae* but compared to CML9, the enhanced resistance observed in CML8 overexpressing lines relies mainly on SA-dependent responses [99]. Emerging data indicate that the regulation of plasmodesmata by plant cells is critical for the establishment of plant defence signalling [102]. Indeed, plasmodesmata are plasma membrane pores that establish cytoplasmic and membrane continuity between cells [102]. It was recently identified that closure of plasmodesmata in response to bacterial flagellin is mediated by the plasmodesmatal-localized CML41 [103]. CML41 is transcriptionally upregulated by PAMPs and facilitates callose deposition at plasmodesmata following flagellin treatment. Using *amiRNA* and *CML41* overexpressing lines, Xu et al. reported that CML41 acts as a positive regulator of defence against *Pseudomonas syringae* [103].

Ca^2+^ signalling is not only required for defence mechanisms upon microbial pathogen attack but also in response to herbivores [58,104,105,106]. Data from Mithoefer’s group indicate that the loss of function of Arabidopsis CML42 enhances resistance to *Spodoptera littoralis* which is correlated with the up-regulation of jasmonic acid-responsive genes and to an accumulation of aliphatic glucosinolates [107]. In contrast to CML42, CML37 acts positively on defence against *S. littoralis* [108]. Indeed, a *cml37* Knock-Out mutant exhibits an enhanced susceptibility to herbivory which is correlated to a lower level of the bioactive form of jasmonate (i.e., JA-Ile) known to be crucial in plant defence coordination against insects [108]. These data suggest opposite roles for CML37 and CML42 in insect herbivory resistance. We cannot exclude that other CMLs such as CML9, CML11, CML12, CML16, CML17 and CML23 could participate in insect defence responses since the corresponding genes are significantly up-regulated in plants treated with oral secretion of the lepidopteran herbivore [109].

Among the range of processes regulated by CaM/CMLs, CMLs are also involved in the suppression of post-transcriptional gene silencing (PTGS), a regulatory mechanism targeting mRNA content [110]. Plant viruses can act both as inducers and as targets of PTGS and this led to the idea that PTGS evolved as a defence mechanism against viruses in plants [110]. Interestingly, a tobacco CML termed rgs-CaM (for regulator of gene silencing) has been reported to act as a suppressor of PTGS [110] and to play a role in antiviral defence by modulating virus-induced RNA silencing [111,112]. The rgs-CaM exerts its antiviral activity by binding and controlling the degradation of viral RNA silencing suppressors. This constitutes the first example of an interaction between a CML and a pathogen protein [111]. Recently, the role of this rgs-CaM in systemic acquired resistance against cucumber mosaic virus has been described in tobacco plants [113]. It was proposed that rgs-CaM functions as an immune receptor that induces salicylic acid signalling by simultaneously perceiving both viral RNA silencing suppressors and Ca^2+^ influx [113].

To date, most of the data describing the involvement of CMLs in different cellular processes associated with plant physiology remain descriptive and new research is now required to decipher the molecular mechanisms controlled by these CMLs. The identification of CML-interacting partners will be crucial to clarify how these CMLs exert their action at the molecular level in plant immunity. For example, CML9, previously described to positively regulate plant immunity, was reported to interact with transcription factors such as the WRKYs and the TGAs, two classes of transcription factors known to play key roles in the regulation of defence processes [69]. PRR2 (*PSEUDO-RESPONSE REGULATOR 2*), a plant specific transcription factor was also described to interact in planta with CML9 but not with the typical CaM [114]. Using a reverse genetic strategy in *A. thaliana*, PRR2 was shown to act as a positive regulator of plant immunity through SA-dependent responses [115].

In many cases, the regulation of these TFs by CML9 or by other CMLs remain presumptive. The next challenge will be to elucidate if all CMLs really act as Ca^2+^ sensors/Ca^2+^ relay proteins, are able to interact with CML-binding proteins and to modulate their activity, as demonstrated for the CaMs. Several questions remain unanswered: Do CaM and CMLs share the same targets? What could be the consequences of such interactions? Interestingly, Yoo et al. showed that the typical CaM (SCaM-1) and the CML (SCaM-4) physically interact with MYB2, a TF that regulates the expression of salt- and dehydration-responsive genes in Arabidopsis [116]. However, SCaM-4 enhances the DNA binding properties of MYB2 whereas SCaM-1 inhibits MYB2 DNA binding [116]. Although these data need validation, different modes of regulation are suggested depending on the interaction with either a CaM or a CML. CaM/CMLs have been reported to interact with different nuclear proteins [68] but do these interactions help to recruit other actors into transcriptional complexes and regulate their activity? What is the contribution of Ca^2+^ in these interactions? Efforts are now made to answer these questions and to better understand the contribution of these sensors in Ca^2+^ signalling with a particular interest on plant specific CMLs.

### 3.3. CDPKs: Positive Regulators of Plant Immune Responses

Beside CaMs and CMLs, plants also possess another class of Ca^2+^ sensors referred to as Ca^2+^ dependent protein kinases or CDPKs. Found only in green algae, land plants and unicellular protists, CDPKs are important Ca^2+^ decoders and relays in plant defence signalling against various types of pathogens (for review: [4]). Their number expanded during evolution of land plants to reach ~30 members in angiosperms (e.g., 34 in *A. thaliana*—29 in *S. lycopersicum*) [117]. The family architecture is nonetheless conserved from mosses to angiosperms, and formed of four distinct groups [22,118]. In contrast to CaMs and CMLs, CDPKs are unique in the repertoire of Ca^2+^ decoders since they combine into a single module Ca^2+^ sensing and downstream signal propagation capabilities. They are composed of a variable N-terminal part, a kinase domain and an activation domain. The activation domain contains an auto-inhibitory pseudo-substrate linked to a CaM-like domain typically containing four EF-hands [119,120]. Upon Ca^2+^ binding by the CaM-like domain, a conformational change displaces the pseudo-substrate from the kinase to allow for downstream phosphorylation events. The dynamic range of Ca^2+^ concentrations that activate a given CDPK therefore depends on the CaM-like domain affinity for Ca^2+^. This likely accounts in part for the specific decoding of different Ca^2+^ variations. Of note, some CDPKs do not show strict Ca^2+^-dependent kinase activity. For example, *Arabidopsis thaliana* CPK7, 8, 13, and CPK30 showed more than half-maximal in vitro syntide-2 phosphorylation activity at very low Ca^2+^ concentrations (1 to 10 nM), with little to no additional kinase activity at higher Ca^2+^ concentrations [121,122]. Whether this reflects true Ca^2+^-independence in vivo or specific Ca^2+^ requirements depending on substrates remains to be clarified [121].

Functional analyses in planta revealed that CDPKs are important components of the plant immune system. Acting alongside and in synergy with the Mitogen Activated Protein Kinases (MAPKs)-dependent signalling cascade, *Arabidopsis* CPK4, 5, 6 and 11 positively regulate defence gene expression upon bacterial flagellin perception [30]. CPK4, 5, 6 and 11 collectively and redundantly contribute to PTI-induced resistance against *Pseudomonas syringae* [30]. CPK5, but also CPK4, 6 and 11, are able to phosphorylate AtRBOHD, the main ROS producing enzyme acting in immunity [122,123]. A model was proposed in which CPK5 and RBOHD sustain a ROS-mediated cell-to-cell communication to reach distal sites from the initial PAMP perception area [124]. Through phosphorylation of the HsfB2a transcription factor, Arabidopsis CPK3 and CPK13 are important regulators of the *PDF1.2* defence gene induction after wounding by the caterpillar *Sodoptera littoralis* [122]. These positive immune regulatory functions of CDPKs prompted their biotechnological use in crop protection against pathogens. For example, overexpression of full length OsCPK4 in rice leads to enhanced disease resistance against *Magnaporthe oryzae* [125]. This enhanced disease resistance in transformed plants is likely due to their higher basal levels of salicylic acid and the resulting potentiation of defence gene induction [125].

In addition to the control of defence gene induction, another important aspect of CDPK function is the control of plant cell death in response to pathogens. Early work on CDPKs in *Nicotiana* sp. showed that CDPK2 and CDPK3 are required for the PCD response triggered after perception of the *Cladosporium fulvum* race-specific Avr4 or Avr9 elicitors [126]. This pioneering work was further expanded in *Arabidopsis* with the demonstration that CPK1 and 2 control the onset of HR upon challenge with avirulent *Pseudomonas syringae* and subsequent NLR-mediated effector recognition [127]. In line with this, several constitutively active CDPKs harbour cell death inducing activity. Auto-active CDPKs (CDPK-VKs) are devoid of their activation domains and therefore do not need Ca^2+^ inputs to be in an active state. Expression of CPK5-VK in Arabidopsis leaf protoplasts leads to cell death and this requires kinase activity [123]. Similar results were obtained in transgenic potato plants stably expressing StCPK5-VK under a pathogen inducible promoter [128]. This latter approach led to increased plant resistance against the hemi-biotrophic oomycete *Phytophthora infestans*. As a trade-off however, plants became more susceptible to the necrotrophic fungus *Alternaria solani* [128]. This contrasted outcome in plant defence toward pathogens with different lifestyles might prevent wider use of these genetic tools in crop protection. PCD induction can also be observed in heterologous expression systems, as demonstrated by the cell death inducing activity of transiently expressed barley CDPK4-VK or Arabidopsis CPK5-VK in tobacco leaves [123,129]. Activation of any given CDPK does not seem sufficient *per se* to provoke PCD since not all CDPKs tested under their auto-active configuration induce PCD [123,129]. The kinase and auto-inhibitory domains of CDPKs being under strong purifying selection [22], it is therefore likely that the specific properties of CDPKs lie, in part, in other features such as their hypervariable termini ends. For example, swapping the N-terminal variable domains of tomato SlCDPK2 and SlCDPK5 exchanged their respective subcellular localizations and their ability to phosphorylate StRBOHB [130]. The existence of cross-species PCD-inducing properties supports nonetheless some degree of functional conservation in CDPKs mode(s) of action through the evolution of land plants.

As already, mentioned, functional redundancy can be problematic when studying particular gene functions within a large family. Although gain-of-function auto-active CDPKs proved of immense value in this context, *cdpk* mutants remain necessary tools and high-order *cdpk* mutants are often required to observe a phenotype of interest such as pathogen susceptibility [30,127]. In contrast to that, a suppressor screen of the *exo70B1* autoimmune phenotype uncovered several *cpk5* loss-of-function alleles [131]. The *exo70B1*-mediated cell death and enhanced resistance to *Golovinomyces cichoracearum* phenotypes were lost in the absence of a functional CPK5, but not of CPK4, 6, or 11 [131]. This advocates for a specific function of CPK5 that cannot be fulfilled here by its otherwise functionally redundant close homologues. In addition to CPK5, the *exo70B1*-mediated autoimmunity also requires the atypical truncated NLR resistance gene TN2 [132]. A tripartite interaction between Exo70B1, CPK5 and TN2 therefore controls cell death. CPK5 kinase activity and its membrane association are both required although the exact mechanism is not completely understood [131]. Whether other CDPKs are also involved in similar interactions, and whether this mechanism represents an innovation present only in *Arabidopsis*, remain open questions.

### 3.4. CDPKs: Also Negative Regulators of Plant Immune Responses

In addition to these positive roles, CDPKs also perform negative functions in plant defence. For example, overexpression of OsCPK12 increased rice susceptibility to avirulent and virulent *Magnaporthe grisae* strains [133] while in barley, overexpressing an auto-active variant of HvCDPK3 increased susceptibility to powdery mildew (*B. graminis* f. sp. *hordei*) [129]. In *Arabidopsis*, perception of several PAMPs by their cognate receptors requires the activity of Somatic Embryogenesis Receptor Kinases 3 (SERK3), a plasma membrane localized receptor and co-regulator also referred to as BAK1 (BRI1-Associated Kinase 1) [134]. In a forward genetic screen set to discover modifiers of the *bak1*–*5* loss-of-function allele [135], Monaghan and colleagues recovered *cpk28* mutants. The loss of CPK28 reverted the impaired ROS production phenotype observed in *bak1*–*5* after PAMP treatments [136]. CPK28 buffers immune responses by modulating the proteasome-mediated turnover of the BIK1 receptor-like cytoplasmic kinase, a shared component of signalling pathways controlled by several PRRs [136]. The search for E3 ligases targeting BIK1 led to the identification of PUB25 and 26, two related E3 ligases of the U-box family whose enzymatic activities towards BIK1 are activated through CPK28-dependent phosphorylation [136]. It is tempting to speculate that the Ca^2+^ influx resulting from PRR activation first participates in ROS production through CPK5 activity, before CPK28 dampens immune outputs. In line with this hypothesis, in vitro assays determined that CPK28 is indeed Ca^2+^ responsive [137]. Interestingly, CPK28 is also able to bind Ca^2+^/CaM, however, this interaction negatively affects its kinase activity in vitro [138]. Ca^2+^ therefore seems to have a dual role in regulating CPK28 activity, thereby, adding another layer of complexity that has yet to be resolved.

Globally, plants have a wide repertoire of Ca^2+^-binding proteins whose functions in plant physiology start to be unravelled. Far from being exhaustive, we have summarized here some examples that describe the importance of certain CaM/CMLs and CDPKs in plant immunity [17,18]. The picture remains nevertheless complex as duplications events and the expected neo-functionalization is sometimes confronted with functional redundancy observed in higher plants. Plants belonging to a sister clade of angiosperms such as liverworts or hornworts could be of interest in discovering yet hidden functions of some calcium sensors. The pursuit of research efforts on these plant specific sensors and particularly the identification of novel CaM/CML-binding proteins and CDPK substrates will be important milestones to get a better view of the many Ca^2+^-regulated events.

## 4. Concluding Remarks and Outlooks

Increasing data support that Ca^2+^, Ca^2+^ sensors and their targets are positioned among the first actors setting up the plant response to biotic interactions. One of the challenges of the coming years will be to understand and expect plant behaviour in a changing environment, with the long-term goal to breed stress-tolerant crops for agriculture. However, although Ca^2+^ signals are the very first step in the signaling pathway, it appears that their manipulation to improve plant resistance could be very challenging. Indeed, calcium signals result from the intricate interaction between pathways and components involved in both their generation and their dissipation such as calcium channels, exchangers or calcium pumps [139]. Nevertheless, some reverse genetic approaches have demonstrated that plant immunity can be modulated through the control of calcium signals by interfering with their generation [140,141] or their dissipation through the activity modulation of calcium pumps [142,143]. Such examples in the literature are rather scarce. Envisioning strategies based on calcium variation modulation to improve plant resistance could be hazardous due to a lack of knowledge concerning actors involved in calcium homeostasis associated to specific plant-microorganism interactions.

An alternative and more amenable approach would be rather to interfere with the function of the Ca^2+^ sensors or their targets that decode Ca^2+^ messages. To date, studies about CaM/CML and CDPKs focus mainly on their interaction with plant target proteins. However, recent and promising data indicate that Ca^2+^ signalling components can be themselves the direct targets of pathogen effectors. In the context of plant immunity, studies on these specifically targeted plant calcium sensors by pathogen effectors might provide a new way for breeding plant resistance. Indeed, it was recently demonstrated that plant CaM is required by the HopE1 effector from *Pseudomonas syringae* to target MAP65, a microtubule-associated protein, to reduce plant immune responses [144]. In a similar way, the *Xanthomonas* AvrBsT effector, able to acetylate ACIP1, a microtubule-associated protein in Arabidopsis and required for both PTI and ETI, possesses a CaM-binding region. AvrBsT is the first member of the YopJ family known to suppress effector-triggered plant immunity [145]. It was reported that AvrBsT is able to interact with CaM in a Ca^2+^-dependent manner and that a mutation of the AvrBsT CaM-binding domain alters or delays the hypersensitive response phenotype (MB Mudgett, personal communication and reports from MPMI meeting 2016 [146]). Whereas CaM has long been known to be a co-factor for mammalian pathogen toxins [147]; these new data obtained with plants support new roles for CaM in plant immunity.

In this review, we focused on Ca^2+^ signalling and biotic stress responses. We point out that CMLs and CDPKs can exhibit a dual function as either positive and/or negative regulators in plant biotic stresses. Moreover, many other data highlight the contribution of Ca^2+^ and Ca^2+^ decoding processes following abiotic stress perception [6,23,63,148]. Studies deciphering plant responses to simultaneously applied abiotic and biotic stress remain sparse. More importantly, these different types of stress are often interrelated in the field and several forms of abiotic stress significantly affect the resistance of plants to bacteria, fungi, viruses and insects [149]. Therefore, is it really attractive to hypothesize that Ca^2+^, Ca^2+^ sensors and their respective targets can be at the crossroads of various signalling pathways and that they could be good candidates to act as central integrators involved in the fine tuning of plant physiological responses to pathogens under fluctuating environmental conditions. As stated in the introduction, it is obvious that Ca^2+^ signalling is not acting as a unique contributor in plant stress responses and we cannot rule out crosstalk between Ca^2+^ and ROS, as well as other second messengers. The next mid and long-term challenges should better characterize the interplay between these signalling pathways.
